# VSM-UNet: A Visual State Space Reconstruction Network for Anomaly Detection of Catenary Support Components

**DOI:** 10.3390/s25195967

**Published:** 2025-09-25

**Authors:** Shuai Xu, Jiyou Fei, Haonan Yang, Xing Zhao, Xiaodong Liu, Hua Li

**Affiliations:** 1 School of Zhan Tianyou, Dalian Jiaotong University, Dalian 116028, China; xushuai@djtu.edu.cn (S.X.); fjy@djtu.edu.cn (J.F.);; 2 School of Electrical Engineering, Southwest Jiaotong University, Chengdu 610031, China

**Keywords:** abnormal detection, catenary support components, deep learning, the state space model (SSMs), attention mechanism

## Abstract

Anomaly detection of catenary support components (CSCs) is an important component in railway condition monitoring systems. However, because the abnormal features of CSCs loosening are not obvious, and the current CNN models and visual Transformer models have problems such as limited remote modeling capabilities and secondary computational complexity, it is difficult for existing deep learning anomaly detection methods to effectively exert their performance. The state space model (SSM) represented by Mamba is not only good at long-range modeling, but also maintains linear computational complexity. In this paper, using the state space model (SSM), we proposed a new visual state space reconstruction network (VSM-UNet) for the detection of CSC loosening anomalies. First, based on the structure of UNet, a visual state space block (VSS block) is introduced to capture extensive contextual information and multi-scale features, and an asymmetric encoder–decoder structure is constructed through patch merging operations and patch expanding operations. Secondly, the CBAM attention mechanism is introduced between the encoder–decoder structure to enhance the model’s ability to focus on key abnormal features. Finally, a stable abnormality score calculation module is designed using MLP to evaluate the degree of abnormality of components. The experiment shows that the VSM-UNet model, learning strategy and anomaly score calculation method proposed in this article are effective and reasonable, and have certain advantages. Specifically, the proposed method framework can achieve an AUROC of 0.986 and an FPS of 26.56 in the anomaly detection task of looseness on positioning clamp nuts, U-shaped hoop nuts, and cotton pins. Therefore, the method proposed in this article can be effectively applied to the detection of CSCs abnormalities.

## 1. Introduction

In the long-term operation of the railroad system, due to the intense vibration and impact during the interaction between pantograph and catenary systems, it is common to see abnormal loosening and loss of the components in the catenary support components (CSCs) and suspension devices. Although the size of these fasteners is relatively small, they play a key role in connecting and fixing all those components, for example, the positioning clamp nuts for fixing the contact wires, the U-shaped hoop nuts for fastening the insulators, and the cotter pins for fixing various components, etc. They need to be promptly identified to avoid jeopardizing the system’s safe operation entire railway.

Anomaly detection technologies based on traditional methods broadly include six aspects: template matching, statistical modeling, image decomposition, frequency domain analysis, classification boundary construction, and sparse coding reconstruction. These methods have been widely used in different application scenarios. For methods based on template matching, Vaikundam et al. [1] used SIFT to extract key feature points and Hough clustering algorithm to find the normal image that best matches the anomalous image as a template for detecting anomalies on steel surfaces. For the methods on statistical modeling, Reed et al. [2] proposed the RX algorithm to calculate the Mahala Nobis distance between checkpoint data and background data to identify anomalies. For the image decomposition, Li et al. [3] combined multi-channel feature extraction with low-rank decomposition algorithms to detect fabric surface defects. For the frequency domain analysis, it includes background spectrum elimination and phase only Fourier transform (POFT) [4]. Among them, the former highlights the anomalous regions by eliminating the spectral information of the background, while the latter tries to eliminate the repetitive background by utilizing only the phase spectrum in the inverse Fourier transform. Sparse coding-based reconstruction methods such as the one proposed by Liang et al. [5] use coding vector sparsity to detect anomalies on touchscreen surfaces. Common methods based on classification surface construction are one-class support vector machines (OC-SVM) [6,7] and support vector data description (SVDD) [8]. The OC-SVM creates hyperplanes in high-dimensional space to separate normal and potentially abnormal samples, while SVDD creates hyperspheres to encompass most normal samples for anomaly detection. These traditional anomaly detection methods are dependent on training samples and often have limitations in terms of generalization and detection speed in practical applications.

In recent years, deep learning has made significant strides in computer vision [9,10,11,12,13,14,15,16]. Compared with traditional methods, deep learning has been widely used in image anomaly detection tasks due to its higher generalizability and the fact that no manual feature engineering is required. Current deep learning anomaly detection methods can be broadly categorized into three types: Distance metric-based, one-class classification-based, and image reconstruction-based. The core idea of these methods based on the distance metric lies in training deep neural networks as feature extractors, so that it can minimize intra-class distances among normal samples and use the distance between test samples and normal features as an anomaly detection measure. Ruff et al. [17] proposed deep support vector data description (Deep-SVDD), where normal samples in the original image space are mapped near the centroids of artificially specified feature points, and accordingly abnormal samples are mapped far away. Deep learning-based distance metric can address the inability of traditional distance anomaly metrics to handle high-dimensional data by converting high-dimensional data into a low-dimensional space. Wu et al. [18] added a decoding module to reconstruct features into images, ensuring that the extracted feature vectors contain enough semantic information and preventing the model degradation issue present in Deep-SVDD. Perera et al [19] introduced the ImageNet dataset to build a classification sub-task aiming to reduce the distance of normal sample features for training purposes, which also has better performance for complex natural image data. The above methods are simple and efficient, but most of them require manually specifying feature centers in advance and designing additional tasks during the training phase to avoid model degradation. Furthermore, they have difficulty to map complex and varied image datasets and data-heavy computations. The core idea of these methods, based on the one-class classification methods, is to construct decision boundaries for the target category, thus distinguish normal and abnormal samples.

Common one-class classification-based methods roughly contain two categories: The former augments the existing dataset by geometric transformations and combines the confidence-based approach of Out-of-distribution detection (OOD) [20] for anomaly detection. The second one considers combining traditional methods like OC-SVM or SVDD to build a classification boundary that fits normal sample distributions as closely as possible to detect anomalies. Golan et al. [21] used 72 geometric transformations based on flipping, translating, and rotating to process the original images, and constructed a classification dataset that contains 72 classes of images. The main idea of these methods based on the image reconstruction method is to map the data to the low-dimensional hidden space, and then reconstruct it backward. Poorly reconstructed data is considered an anomaly. It mainly includes the ideas of autoencoder (AE) [22] and generative adversarial network (GAN) [23]. For example, in GANomaly [24], the model is first trained to achieve the reconstruction of normal images from encoding to decoding, and then calculates pixel-level anomaly maps based on the reconstruction error. Sudao He [25] tries multimodal feature description network and prompt-aided cross-modal graph learning algorithm to identify the defects contours. A new neighbor mask block coverage strategy and dynamic weight feature domain adaptive alignment method is proposed to analyze the low-illumination catenary component [26,27]. 

In recent advancements in industrial anomaly detection, distance-based methods require manual specification of feature centers, posing challenges when handling complex, large-scale datasets. In the domains of one-class classification and image reconstruction, researchers commonly employ encoder–decoder architectures composed of Convolutional Neural Networks (CNNs) and Transformers, which are primarily utilized for detecting significant anomalous features (CNN), yet are less sensitive to subtle variations. Furthermore, both CNNs and Transformers face limitations: CNNs exhibit weak long-range feature correlation capabilities, while Transformers suffer from high computational complexity. As a result, these methods struggle to fully leverage their performance when applied to the detection of minute anomalies, such as loosening or missing components in catenary systems. Fortunately, VMamba, based on state-space models (SSMs) [28], not only effectively associates long-range features within feature maps through a continuous scanning mechanism, enhancing feature extraction performance, but also offers a more compact parameterization and reduced model complexity compared to similarly scaled Transformer models. Moreover, with a topology akin to Swin-Transformer, VMamba integrates effectively with attention mechanisms, amplifying the model’s sensitivity to minor anomalies [29,30,31,32,33,34]. 

Inspired by the above analysis, the present work proposes a novel Visual State-Space Reconstruction Network (VSM-UNet), built upon state-space models, for detecting loosening anomalies in catenary components [35,36,37,38,39]. The specific contributions are outlined below.

- Based on the UNet structure, a new “visual state space reconstruction network (VSM-UNet)” is constructed using visual state space blocks (VSS blocks) and CBAM attention mechanisms for detecting loosening anomalies of CSCs.
- An anomaly score calculation module based on MLP network is designed to help the model evaluate anomaly levels. Then, a new loss function is proposed to assist model training and improve the anomaly score calculation module.
- The effectiveness of this method is verified on the abnormal CSCs dataset of positioning clamp nuts, U-shaped hoop nuts, and cotter pins.

## 2. The Proposed Methods

The relevant fasteners of the catenary support devices in electrified railways are typically composed of small parts such as nuts, bolts, and cotter pins. When these fasteners experience loosening or deformation anomalies, the changes are usually subtle, which creates a performance bottleneck for classical dynamic template anomaly detection methods. In addition, most existing anomaly detection methods rely on single-form CNN or Transformer models, which perform poorly in long-range feature correlation extraction and computational complexity. To address these issues, we introduce the Convolutional Block Attention Module (CBAM) and the VMamba module into the UNet detector and design an anomaly scoring module to assess fastener anomalies. Specifically, this paper proposes a new Visual State-Space Reconstruction Network (VSM-UNet) detector. In this section, we first provide a detailed introduction to the basic modules in VSM-UNet (VSS modules and CBAM). Then, the article outlines the main structure of the training method. Finally, we describe the learning strategies used during training.

### 2.1. Visual State Space Block (VSS Block)

**Figure 1:**
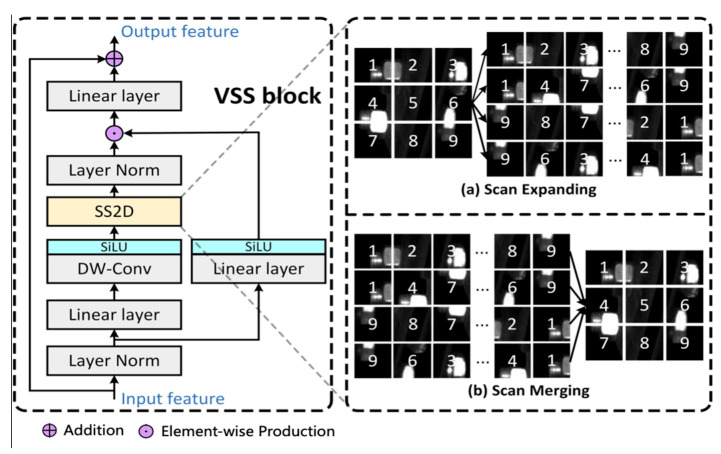
The structure of VSS Block.

In this paper, we adopt VSS block as the feature extraction core module of the VSM-UNet. The VSS block is illustrated in Figure 1, which is mainly based on VMamba [28]. Specifically, the conventional State Space Sequence Models are linear time-invariant system functions. It maps *x*(*t*) ∈ R into *y*(*t*) ∈ R through a hidden state *h*(*t*) ∈ R*^N^*. Among them, *A* ∈ R*^N^*^×*N*^ is used as the evolution parameter; *B*, *C* ∈ R*^N^*^×1^ is used as the projection parameters for a state size *N*; the skip connection parameter is set to *D*. The model can be expressed as a linear ordinary differential equation (ODE) as follows.

(1) $$\begin{array}{c} h^{\prime }(t)=Ah(t)+Bx(t)\\ y(t)=Ch(t)+Dx(t) \end{array}$$

The linear model can be discretized by a zero-order transformation preserving the given time scale parameter Δ ∈ *R^D^*.

(2) $$\begin{array}{c} h_{t}=\bar{A}h_{k-2}+\bar{B}x_{k}\\ y_{t}=Ch_{k}+\bar{D}x_{k}\\ \bar{A}=e^{\Delta A}\\ \bar{B}=(e^{\Delta A}-I)A^{-1}B\\ \bar{C}=C \end{array}$$

where *B*, *C* ∈ R*^D^*^×*N*^. We derive an approximation of $\bar{B}$ using the first-order Taylor series$\;\bar{B}=(e^{\Delta A}-I)A^{-1}B\approx (\Delta A){\left(\Delta A\right)}^{-1}\Delta B\;=\Delta B$. Next, the Visual Mamba further integrates the Cross-Scan Module (CSM) and convolution operations into the block [28]. In the VSS block, the feature flow first passes through the linear embedding layer, and then bifurcates into dual pathways. The feature flow in a branch first undergoes depth-wise separable convolution [40] and SiLU activation functions; then, the feature flow is introduced into the SS2D module and layer normalization module; finally, it merges with the feature flow from another branch that passes through a linear layer and SiLU activation function. Unlike typical visual Transformers, this VSS block omits positional embedding operations and ops for streamlined structures without an MLP stage, resulting in denser block stacking within the same depth budget. Note that SS2D includes ‘Scan expanding’ and ‘Scan merging’, as shown in the figure below.

### 2.2. Convolutional Block Attention Module (CBAM)

**Figure 2:**
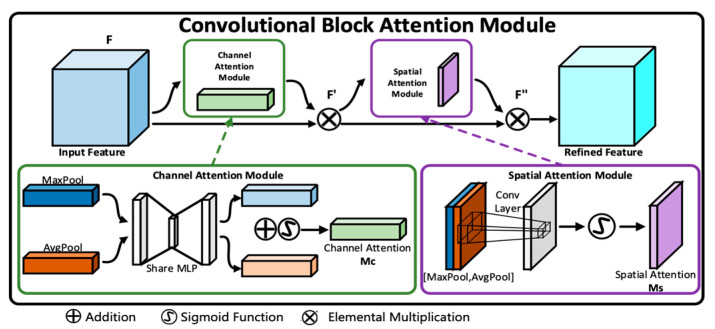
The structure of CBAM.

The convolutional block attention module (CBAM) is a simple, effective, and plug and play attention module. As shown in Figure 2, for a given intermediate feature map input, the module infers attention maps along two independent dimensions (channel and spatial spatial) in order. The calculation question can be written as follows [41].

(3) $$\begin{array}{c} F^{\prime }=M_{c}\left(F\right)⊗F=\left[\sigma_{2}\left(MLP\left(AvgPool\left(F\right)\right)+MLP\left(MaxPool\left(F\right)\right)\right)\right]⊗F\\ F^{\prime \prime }=M_{s}\left(F^{\prime }\right)⊗F^{\prime }=\left[\sigma_{2}\left(f^{7\times 7}\left(\left[AvgPool\left(F^{\prime }\right);MaxPool\left(F^{\prime }\right)\right]\right)\right)\right]⊗F^{\prime } \end{array}$$

where ⊗ denotes elemental multiplication; *F*′ represents the output of channel-attention feature mapping; *F*″ is the output of channel-spatial attention feature mapping; *M_s_* ∈ R^1×*H*×*W*^ is the 2D spatial attention feature mapping; *M_c_* ∈ R*^C^*^×1×1^ is the 1D channel attention feature mapping; *MLP* denotes multi-layer perceptron; *AvgPool* is the average pooling operation; *MaxPool* denotes the maximum pooling operation; *f*^7×7^ denotes convolution with kernel size 7 × 7; *σ*_2_ is the Sigmoid activation function.

### 2.3. Visual State Space Reconstruction Network (VSM-UNet)

**Figure 3:**
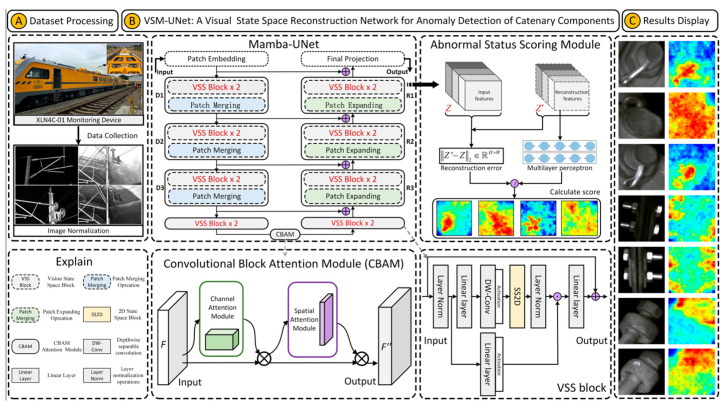
The structure of VSM-UNet.

In Figure 3, the overall architecture of VSM-UNet is presented. The architecture is inspired by UNet [42] and Swin-UNet [43]. Specifically, the VSM-UNet includes a patch embedding layer, multi-layer encoder, multi-layer decoder, final projection layer, skip connections, CBAM, and abnormal status scoring module. Among them, multi-layer encoder consists of VSS block and patch merging; multi-layer decoder includes VSS block and patch expanding operation; the anomaly score scoring module consists of reconstruction error and multi-layer perceptron (MLP). It is worth noting that, unlike Swin-UNet, we did not use a symmetrical structure but instead adopted an asymmetric design. The architectural overview of VSM-UNet is as follows.

First, the input image is divided into non-overlapping patches with a size of 4 × 4. Then, the dimension of the image is mapped to C, which defaults to 96. It can be expressed as the following formula:

(4) D=Patch_embedding(Z)

where *Z* ∈ R*^H^*^×*W*×1^ denotes 2D grey-scale image; Patch_embedding denotes patch embed ding operation; *D* ∈ R^(*H*/4)×(*W*/4)×*C*^ represents the embedded image. Then, before inputting *D* into the encoder for feature extraction, we use a layer normalization (LN) operation to normalize it. Next, the multi-layer encoder consists of four stages. The encoder reduces the height and width of the input features while increasing the number of channels through a patch merging operation at the end of the first three stages. The process of feature encoding is as follows:

(5) H=Mencoder(D)

where *H* ∈ R^(*H*/32)×(*W*/32)×8*C*^ presents the final encoder feature; *Mencoder* denotes a level by level encoding operation. It is worth noting that we used [2, 2, 2, 2] VSS blocks in four stages and set the number of channels for each stage to [*C*, 2*C*, 4*C*, 8*C*]. Then, we used CBAM attention module to enhance the weight of important information in the encoded feature, thereby optimizing the anomaly detection effect.

(6) Att=CBAM(H)

where *Att* ∈ R^(*H*/32)×(*W*/32)×8*C*^ represents the attention weighted output of the encoding layer; *CBAM* denotes the CBAM attention module. Similarly, the decoder is organized into four stages. At the beginning of the last three stages, we used a patch expanding operation to reduce the number of feature channels and increase their height and width. The above feature decoding (reconstruction) process is as follows:

(7) R=Mdecoder(Att)

where *R* ∈ R^(*H*/4)×(*W*/4)×*C*^ represents the reconstruction feature from the decoder; *Mdecoder* denotes a level by level encoding operation. In these four stages, we used [2, 2, 2, 1] VSS blocks with channel counts of [8*C*, 4*C*, 2*C*, *C*]. After using the decoder, we used the final projection layer to recover the size of the reconstruction features for calculating the anomaly score of the image. Specifically, we first perform four up-sampling operations through patch expanding to recover the height and width of features, and then use a projection layer to recover the number of channels [28]:

(8) $$Z^{\prime }=Projection\_layer(R)$$

where *Z*′ ∈ R*^H^*^×*W*×1^ represents reconstructed feature map with input size; Projection_layer denotes final projection layer. For the skip connections, a sample add operation is used without any prompts, so no additional parameters are introduced into the models. Finally, to better refine the reconstruction error, we use a multi-layer perceptron to adjust the anomaly score map. The adjustment factor is calculated as below:

(9) $$\Delta =MLP(Z^{\prime })$$

where Δ ∈ R*^H^*^×*W*^ denotes scale-factor for adjusting reconstruction error; *MLP* represents the multi-layer perceptron.

### 2.4. Learning Strategy and Anomaly Score Calculation

Mean Square Error (MSE) represents the mean square error of each element between the predicted quantity and the target quantity. It is often used as a loss function in regression tasks. In addition, the MSE function curve is smooth, continuous, and differentiable everywhere. During gradient descent, the gradient decreases as the calculation error value decreases, which helps the model to converge quickly. Therefore, this paper used MSE as the reconstruction loss to train the encoding–decoding part of the VSM-UNet network. Its calculation formula is as follows:

(10) $$L_{UN\mathrm{et}}=MSE(Z,Z^{\prime })$$

where the reconstruction error ||*Z*′ − *Z*||_2_ is used for calculating the anomaly score map; L_UNet_ denotes anomaly score map mean square error of VSM-UNet network; ||⋅||_2_ represents *l*_2_ norm across channels; *MSE* denotes mean square error loss function. It is worth mentioning that there are still differences between normal feature maps and generated feature maps. To solve the problem, we used an MLP network to estimate the score variance. Specifically, the output of the *MLP* (Δ ∈ *R^H^*^×*W*^) is used as a scale-factor to refine the reconstruction error map into an anomaly score map. Then the final score map *S* ∈ R*^H^*^×*W*^ can be defined as:

(11) $$S={\left\|Z^{\prime }-Z\right\|}_{2}Ø\Delta$$

where Ø denotes an element-wise division. The MSE loss *L_MLP_* is used to train and optimize the MLP network. Its calculation method is as follows.

(12) $$L_{MLP}=MSE(\Delta ,||Z^{\prime }-Z|{|}_{2})$$

where *L_MLP_* denotes *MLP* training loss function. Then the total loss function *L_total_* is defined as follow.

(13) $$L_{Total}=L_{UNet}+L_{MLP}$$

It should be noted that to better optimize these two parts, the gradient flow between Mamba-UNet and MLP is truncated.

## 3. Experimental Results and Analysis

This section mainly introduces the dataset, experimental details (environmental settings, experimental parameters, evaluation indicator, experimental steps) and experimental analysis.

### 3.1. Description of Experiments

#### 3.1.1. Experimental Data and Parameter Settings

**Figure 4:**
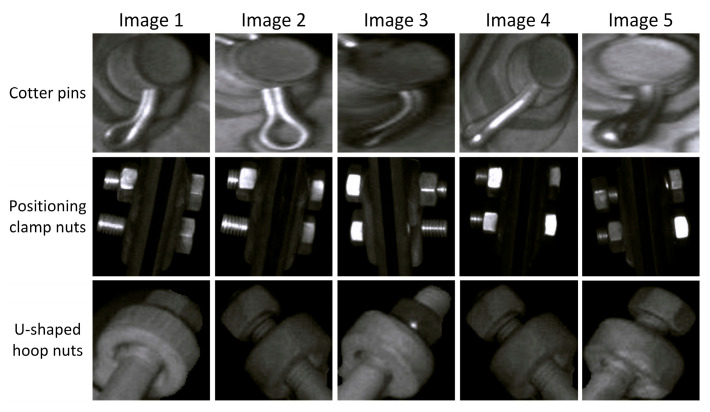
Examples of partial component images.

The data collected in this experiment are global images of the catenary support components, which were taken by a high-speed rail inspection vehicle along a certain railway line (the original images collected include a total of 6151 pictures). We used 5226 selected images with clear quality and meeting the requirements to construct the data set for this experiment, as shown in Figure 4. It is worth mentioning that in actual working conditions, more than half of the abnormal conditions (such as loosening) of contact network support components often occur in cotter pins, positioning clamp nuts, U-shaped hoop nuts, and other parts. To this end, we employed the YOLOv8 object detection algorithm [44] to extract regional-level images of the three components and selected a portion of samples for anomaly detection. The distribution of the extracted regional-level fastener samples is shown in Table 1. Specifically, we first selected 1000/1000/850 normal samples of positioning clamp nuts, U-shaped hoop nuts, and cotter pins, respectively, for the training set. Next, for each type of fastener, 300 normal samples were selected as positive samples for the test set. Finally, since the abnormal samples (negative samples) were relatively scarce, we applied data augmentation (rotation, flipping, translation) and randomly selected 125, 146, and 173 images, respectively, for inclusion in the test set.

The hardware and software configuration of this experiment are as follows: (1) CPU: Intel(R) Core (TM) i9-14900K 3.20 GHz; (2) Operating memory: 64G RAM; (3) GPU: NVIDIA RTX 4090 GPU; (4) Operating system: Ubuntu 18.04; (5) Code operating environment: Pytorch = 2.2.1, Python = 3.10; (6) CUDA version: CUDA 12.3, cuDNN 8.9.7. In addition, the specific hyperparameter settings for the experiments are shown below: (1) Data augmentation: filling the image to 1.2 times the original size, randomly rotate 10 degrees, and randomly crop to the original size; (2) Training settings: the training process uses the Adam optimizer, whose Beta parameters are set to (0.5, 0.999); (3) the training period, learning rate and batch size are set to 100 epochs, 0.0001, and 128.

#### 3.1.2. Experimental Evaluation Index

The defect detection model determines whether test samples are normal or defective, which is essentially a two-classification problem. Therefore, classification evaluation indicators such as recall rate, precision rate, and F1 score are more suitable. In the experiment, defective samples are regarded as positive samples, so TP, TN, FP, and FN respectively correspond to successfully identified defective samples, correctly classified normal samples, normal samples mistakenly identified as defects, and missed defective samples. Correspondingly, a higher recall rate indicates that defective samples are less likely to be missed, and a higher precision rate indicates that normal samples are less likely to be falsely detected.

In addition, in the field of anomaly detection, the Area Under the Receiver Operation Feature Curve (AUROC) is also an important basis for evaluating model detection performance. AUROC represents the area under the receiver operating characteristic curve [45] (Receiver Operation Feature Curve, ROC). The ROC curve uses the predicted probability of each test sample as the threshold in turn, with the false positive rate (False Positive, FP) as the abscissa and the true positive rate (True Positive Rate, TP) as the ordinate. It can record the performance of binary classification models under all threshold conditions. The closer the curve is to the upper left, the better the performance. The calculation formulas of FP and TP are as follows.

(14) $$\begin{array}{l} FP=\frac{FP}{FP+TN}\\ TP=\frac{TP}{TP+FN} \end{array}$$

AUROC is the area under the entire ROC curve between coordinates (0, 0) and (1, 1), which measures the potential of the model to correctly classify samples. When the value of AUROC is 1, the model is an ideal classifier; when AUROC = 0.5, it means that the prediction result is equivalent to random guessing; when the value of AUROC is between 0.5 and 1, it indicates that it has certain predictive value, and the larger the value within this range, the more likely the model is to obtain better classification results through appropriate thresholds.

### 3.2. Experimental Analysis

#### 3.2.1. The Analysis of the Impact of Background Suppression on Detection Results

**Figure 5:**
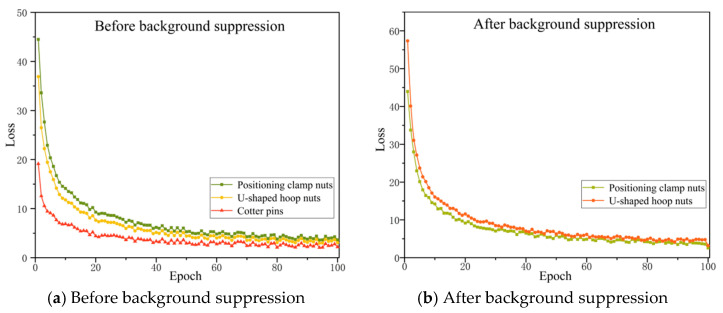
Loss function curves of original UNet on different components.

To verify the improvement effect of background suppression on loosening fault identification, this experiment conducted a background suppression experimental analysis on the positioning clamp and U-type hoop nut and used them to train the original UNet model (SAM segmentation algorithm was used for background suppression). The cotter pins are mostly found on catenary parts such as insulator bases and binaural lugs. There are many phenomena such as small scale, relatively uniform background, and low noise interference. Therefore, the background suppression effect will not be discussed. The loss function curve of the training process is shown in Figure 5. It can be seen from the figure that during the training cycle, the model loss continues to decrease and finally converges, and the model is basically fitted at the 100th epoch.

At the end of training, the weight with the smallest loss value is first saved as the best model for this training, and then the best model is used to test the test sample, and finally the anomaly score map of the test image is obtained. Furthermore, we used the maximum value in the anomaly score map as the anomaly score of the corresponding test image to characterize the abnormality degree of the sample. The AUROC values of each component of the original UNet model are finally obtained and are shown in Table 2.

**Figure 6:**
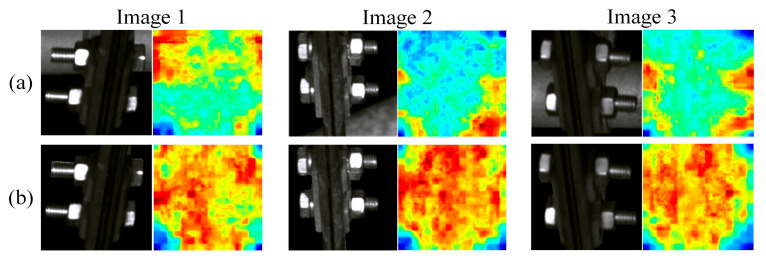
Comparison of abnormal score map of normal positioning clamp nuts ((**a**) before background suppression, (**b**) after background suppression).

As can be seen from the table, after background suppression, the AUROC values of the original UNet for positioning clamp nuts and U-shaped hoop nuts increased by 28.1% and 8.2%, respectively; background suppression improves the detection performance (AUROC value) of VSM-UNet on positioning clamp nuts and U-type hoop nuts by approximately 24.8% and 4.9%; compared to the original UNet, VSM-UNet has an advantage of about 1.8%~6.8% in AUROC performance. This not only proves that background suppression can effectively improve the fault detection effect, but also proves the superiority of VSM-UNet over UNet. To demonstrate the improvement effect of background suppression more intuitively on detection, we visualized the abnormal score map of normal samples in the localization line clamp test set in the form of a single normalized heatmap and presented it in Figure 6.

For the three samples in the figure, the first line is the image before background suppression and its anomaly score map, and the second line is the image after background suppression and its anomaly score map. Before background segmentation, due to the large intra-class differences of normal samples, the score of complex background areas is significantly higher than that of component areas, which increases the risk of being misidentified as faults. After background segmentation, without complex background interference, the abnormality degree of each area of the normal sample is more consistent. This is helpful to widen the anomaly score gap with fault samples, thereby improving the AUROC value of the model. Therefore, we used segmented images as input in subsequent experiments.

#### 3.2.2. Comparison with Other Fault Detection Methods

To fully prove the advantages of this method in detecting loose parts anomalies, this experiment selected other classic anomaly detection methods: SSIM-AE [46], Trust-MAE [47], GANomaly [24], and DRAEM [48], for performance comparison. These methods are representative in reconstruction-based anomaly detection. In the data set shown in Table 1, the comparison method is trained using the network parameters and training process recommended by the author of the original paper. The AUROC, FPS, Recall, Precision, and F1-Score are used as evaluation indicators of detection performance to evaluate the anomaly recognition effect and inference speed of the model, respectively. The average test results of the three types of components are shown in Table 3.

As can be seen from Table 3, the AUROC, Recall, Precision and F1 Score of this method are significantly higher than the other four models by about 10.2% to 18.4%, and higher than the DRAEM model with the best comprehensive performance by about 10.2% to 11.3%. This shows that the algorithm has obvious advantages in accuracy of identifying abnormal looseness of catenary components. In terms of detection speed, the DRAEM model has obvious advantages. In comparison, the method in this paper is inferior in detection speed (FPS = 26.56). Fortunately, in actual catenary state detection, the system places more emphasis on the requirement of recognition accuracy. Therefore, considering the accuracy and inference speed of anomaly recognition comprehensively, the method proposed in this paper has significant advantages in practical engineering applications.

#### 3.2.3. The Analysis of Attention Mechanism

We set up the CBAM module in VSM-UNet to enhance the model’s ability to pay new attention to key features. To select the appropriate attention mechanism, we conducted experimental analysis on some classic non-hybrid attention mechanisms such as SE block (SE) [49], Non-local (NL) [50], and hybrid attention mechanisms such as Dual-Attention (DA) [51] and CBAM [41]. The results are shown in Table 3. It should be noted that the AUROC value in the table is the average of the detection effects of VSM-UNet on three types of components.

The following results can be drawn from the results in Table 4: (1) The comprehensive evaluation index of hybrid attention (DA, CBAM) is better than the non-hybrid attention mechanism method (SE, NL); (2) CBAM has better performance in Recall, Precision, and F1-score, and the average AUROC indices are better than DA. Therefore, this article chooses CBAM as the attention feature enhancement module of VSM-UNet.

#### 3.2.4. The Analysis of Anomaly Detection Visualization Effects

The above experiments confirmed the effectiveness of background suppression processing, so background suppression methods were adopted in subsequent experiments. Table 4 has shown that the model has achieved considerable results on image-level indicators. Next, the fault location effect of the model will be verified. For rupture defects, many research experiments compute pixel-level metrics via hand-crafted mask labels. However, looseness faults mainly manifest themselves as abnormal logical relationships and spatial positional relationships between component elements. Furthermore, it is difficult to calibrate the abnormal area in the image to a precise range, so the pixel-level indicators cannot accurately reflect the positioning effect of loose faults. Therefore, this experiment demonstrates the fault location effect of this method in the form of anomaly score heat map.

**Figure 7:**
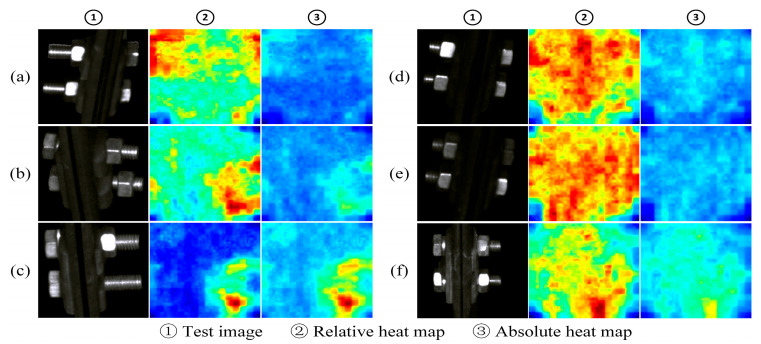
The localization effect of positioning clamp nuts ((**a**) normal components, (**b**) loosened faulty components, (**c**) missing faulty components, (**d**–**f**) are normal samples).

As shown in Figure 7, the figure shows the fault location effect of some samples in the positioning clamp nuts test set. Each sample has two corresponding heat maps. These two heat maps, respectively, represent the normalized representation of anomaly scores on a single image (relative heat map) and the normalized representation of anomaly scores on the entire test set level (absolute heat map). The relative heat map mainly reflects the difference in abnormality between pixels within a sample, while the absolute heat map reflects the abnormality ranking of the pixels in the sample in the overall test set.

The samples (a), (b), and (c) in the figure are faulty samples, and the nuts are loosened in order of severity. From slightly loose to completely missing, the faulty nut shows an abnormality score higher than the normal area, and the faulty area is located. Among them, the reflection of the gasket on the upper left side of sample (c) leads to a higher score in this area, but there is still a certain gap compared with the real fault area. The samples (d), (e), and (f) in the figure are normal samples, and the relative heat maps of (d) and (e) have large and scattered high score areas. This shows that the abnormality degree of each pixel is relatively consistent. It can be seen from its absolute heat map that the overall score of the sample is low, and no obvious abnormal areas are identified. In sample (f), the lower portion of the part is highlighted due to reflection. This results in a cluster of high scores just below both heatmaps, indicating that the sample was misidentified as a minor anomaly.

**Figure 8:**
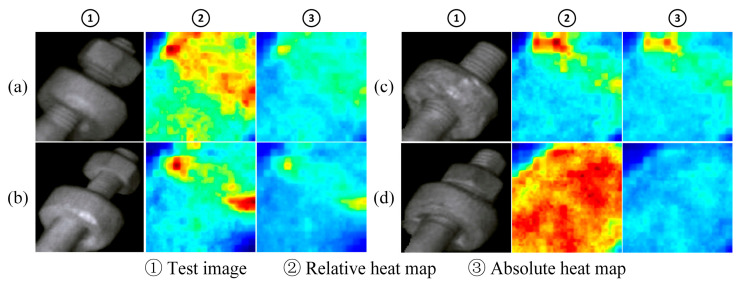
The localization effect of U-shaped hoop nut failure ((**a**)–(**c**) the different faulty components. (**d**) the normal component).

Figure 8 shows the fault location effect of some samples in the U-type hoop nut. The composition of each group of pictures is consistent with that in Figure 7. The samples (a), (b), and (c) in the picture are faulty samples, and the degree of nut loosening is different. It can be seen from both the relative heat map and the absolute heat map that for each degree of looseness, the area around the faulty nut shows significantly higher scores, indicating that the fault area is roughly identified. For the normal sample (d), the high-scoring area in its relative heat map accounts for a large proportion of the entire image, while the overall score in the absolute heat map is low, which indicates that no obvious abnormal area has been identified.

**Figure 9:**
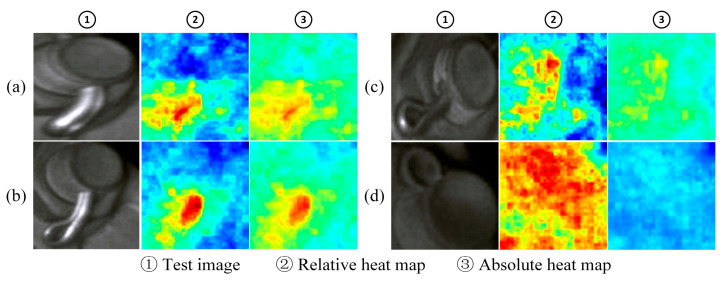
The localization effect of cotter pin failure ((**a**)–(**c**). the different faulty components. (**d**) the normal component).

Figure 9 shows the fault location effect of some samples of cotter pins. Among them, sample (d) is a normal sample, while the rest are faulty samples, and the degree of looseness of the cotter pin decreases from sample (a) to (c). As observed in the relative heat map, the high-scoring areas of the fault sample are concentrated near the protruding cotter pin, which is significantly different from the scores of other normal areas. The high-scoring areas of normal samples are distributed over a wide range. It can be seen from the absolute heat map that the overall score of the fault sample is higher, and as the degree of looseness increases, the score at the cotter pin gradually increases. The overall score of the normal sample is very low, which is significantly different from the fault sample.

The above examples show that the model in this paper performs better in locating loose faults in most samples. However, when the parts have large differences in shape (e.g., parts with complex shapes, such as reflective nuts and gaskets, insufficient lighting, etc.), the model will still have detection errors.

#### 3.2.5. The Analysis of the Impact on Score Processing Methods and Thresholds on Anomaly Recognition Results

The anomaly score map reflects the anomaly score of each pixel in the map, and the fault areas of different types of components have different shapes and sizes. How to obtain the most appropriate score value that can characterize the abnormality of the entire image is one of the issues to be discussed in this experiment. To verify the impact of different score processing methods on fault identification effects, this experiment uses different data processing methods. Specifically, the maximum value in the anomaly score map, and the maximum value and average value after average pooling are, respectively, taken as the image-level anomaly score. The AUROC value is shown in Table 5.

The ‘4 × 4’ in the third row of the table indicates that before calculating the maximum, average pooling is performed on the score map using a kernel size of 4. Similarly, the last four rows indicate that kernels of sizes 16, 32, 64, 72, and 128 are used to perform average pooling on the score map before calculating the maximum value. The optimal anomaly score calculation methods for different types of parts are different. After using the best data processing method for all three parts, the average AUROC values reached 0.978.

For U-type hoop nuts and pins, the failed nuts and cotter pins account for a large proportion of the entire image (the area with high abnormality score is larger), so using a processing method with a high average degree can obtain a higher AUROC value. Among them, the U-shaped hoop nut has the largest proportion of high-scoring areas, and the average value of the entire picture is the most representative. The proportion of cotter pins in the pins is slightly smaller, so it is most appropriate to use an average pooling operation with a kernel of 128 × 128 and then find the maximum value as a representative. For the positioning clamp, the failed nut occupies a smaller proportion of the entire picture than the above two components. Therefore, the maximum value obtained after using average pooling with a core of 64 × 64 to reduce noise interference is the most representative of the entire image.

**Figure 10:**
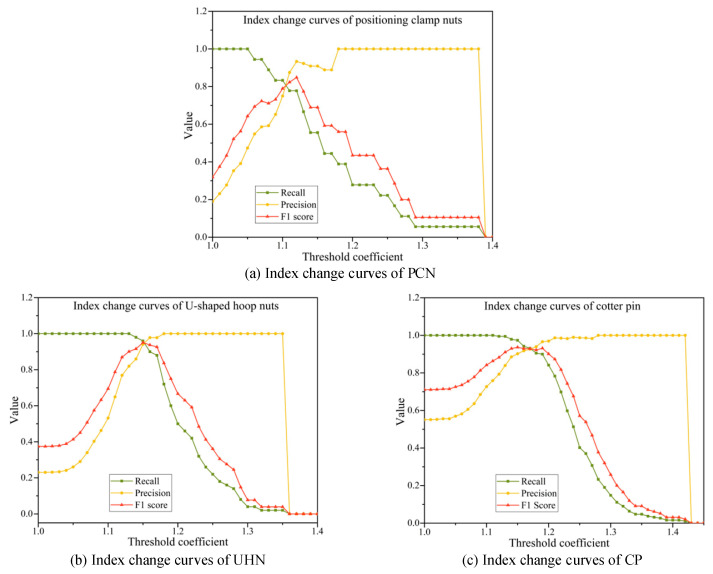
Change curve of classification evaluation index with threshold coefficient.

After determining the score value that best characterizes the abnormality of the entire image, the impact of the score threshold on the fault identification effect is another issue to be discussed in this experiment. This experiment uses the anomaly scores of the training set samples as the basis for threshold setting. Considering that the model has learned the training set samples, the abnormal scores of the training set samples will be smaller than the normal samples of the test set. Therefore, this paper uses the result of multiplying the average anomaly score of the training samples by the amplification factor a as the threshold. In this experiment, the threshold coefficient is assigned a value between 1 and 1.3 at an interval of 0.01. The values of the three evaluation indicators of recall, precision, and F1 score of the test set sample change curve with the threshold coefficient, as shown in Figure 10.

The following conclusions can be seen from the figure: (1) As the threshold coefficient value increases, the recall rates of the three types of parts gradually increase to 1; (2) The precision rates gradually decrease from 1; (3) The F1 scores all show a trend of rising first and then falling and reach a maximum value near the intersection of recall rate and precision rate. This shows that the threshold setting has a great impact on the results of model fault identification. A small threshold can ensure that fault samples are not missed, but many normal samples will be mis-detected. A large threshold can reduce false detections, but missed detections will increase at the same time. Considering using the comprehensive indicator F1 score to balance recall and precision, we choose the threshold with the highest F1 score as the optimal threshold to ensure that both recall and precision are at a good level. It can be seen from the figure that the optimal threshold coefficients of the positioning clamp nuts are approximately 1.12, respectively, and the optimal threshold coefficients of the U-shaped hoop nuts and cotter pin are both approximately 1.15 and 1.18. Under this optimal threshold, the statistics and performance indicators of the two-class classification results of the three components are shown in Table 6, respectively.

The experimental results indicate that the recall rate for positioning clamp nuts is relatively low (77.6%), primarily due to the limited number of fault samples and the higher proportion of missed detections. This highlights the model’s limitation in anomaly recognition under small-sample conditions. However, its precision is relatively high (97.0%), demonstrating that the identified anomalies are highly reliable. In contrast, U-shaped hoop nuts achieve balanced performance across recall, precision, and F1 score (all at 95.9%), showing stable detection capability and the best overall performance, which underscores its strong engineering application value. Cotter pins exhibit the highest recall rate (97.1%), nearly covering all abnormal samples, but their precision is lower (81.2%), indicating a higher rate of false detections. This suggests that the model tends to prioritize avoiding missed detections for this component, albeit at the cost of reduced accuracy. Overall, the proposed method demonstrates strong effectiveness across different component detection tasks, particularly in recall performance, validating its feasibility and application potential in abnormal detection of electrified railway catenary components.

#### 3.2.6. The Analysis of Ablation Experiments

To verify the effectiveness of the improved strategy in VSM-UNet, we conducted the following ablation experimental analysis. Among them, (-) VSS Block means replacing the VSS block, Patch merging, and Patch expanding modules in VSM-UNet with the original UNet module; (-) CBAM means ablation of CBAM; (-) None means no ablation operation is performed. The experimental results are shown in Table 7.

From the results shown in Table 7, the ablation of the CBAM module and the VSS block has a negative impact of about 1.1~1.2% on the classification index (Recall, Precision, F1 score, and AUROC) of the proposed method. Among them, the negative impact of VSS block ablation is greater than that of CBAM. The possible reasons are as follows: (1) The VSS block module is the main feature extraction module in VSM-UNet, and its feature optimization capabilities can bring significant performance improvements to the model; (2) Compared with convolutional networks, the visual state space extraction module (SSM) in the VSS block can better improve the model’s spatial information capture capability. It can be seen from the FPS results that the introduction of VSS block and CBAM module will reduce the inference speed of the model (about 5.1–8.76 FPS). Fortunately, this reduction in reasoning speed is small and acceptable.

## 4. Conclusions

This paper mainly conducts research on loosening fault detection of catenary support com ponents. First, we constructed a loose fault detection data set of positioning clamps nuts, U-shaped hoop nuts, and cotter pins. Then, based on UNet and state space model (SSM), an algorithm specifically used for abnormal detection of loosening on catenary support parts was constructed. The design of this algorithm mainly has three aspects. First, it uses VSS block, Patch merging, and Patch expanding to replace the encoding–decoding convolution block in the original UNet. Then, the CBAM attention module is embedded after the encoder of the model to enhance key features and weaken interfering features. Finally, MLP is used to calculate anomaly score maps, and the fault classification results under multiple threshold values are compared to determine the optimal fault discrimination threshold, thereby effectively identifying three types of component loosening faults. In addition, through performance comparison with other advanced anomaly detection methods, we have verified that the proposed method has significant advantages in detecting loose faults in catenary support components. Next, we will consider how to further improve the accuracy of the detection method in cases where the shape consistency of components is poor, as well as how to employ contrast enhancement techniques [52] to further enhance the detection performance of fasteners.

## Figures and Tables

**Table 1:** The statistics of experimental data.

Categories	Training Set	Testing Set	Testing Set
	Normal Samples	Normal Samples	Abnormal Samples
Positioning clamp nuts	1000	300	125
U-shaped hoop nuts	1000	300	146
Cotter pins	850	300	173
Total samples	2850	900	444

**Table 2:** The analysis of background suppression with AUROC value.

Categories	Original UNet		VSM-UNet	
	Before Suppression	After Suppression	Before Suppression	After Suppression
Positioning clamp nuts	0.647	0.928	0.715	**0.963**
U-shaped hoop nuts	0.865	0.947	0.927	**0.976**
Cotter pins	0.948	—	**0.966**	—

**Table 3:** The results of comparative experiments.

Methods	Recall	Precision	F1-Score	AUROC	FPS
SSIM-AE	0.719	0.746	0.731	0.8	30.66
Trust-MAE	0.748	0.773	0.767	0.831	31.76
GANomaly	0.741	0.757	0.756	0.819	27.42
DRAEM	0.796	0.817	0.807	0.871	**61.28**
Ours	**0.904**	**0.928**	**0.916**	**0.981**	26.56

**Table 4:** The results of attention mechanism.

Methods	Recall	Precision	F1-Score	AUROC
SE	0.89	0.914	0.902	0.965
NL	0.885	0.908	0.896	0.962
DA	0.898	**0.927**	0.912	0.974
CBAM	**0.902**	0.926	**0.914**	**0.979**

**Table 5:** Analysis of AUROC values under different anomaly score calculation methods.

Processing Method	Positioning Clamp Nuts	U-Shaped Hoop Nuts	Cotter Pins
Max value	0.967	0.979	0.959
4 × 4	0.969	0.981	0.96
16 × 16	0.968	0.983	0.959
32 × 32	0.966	0.984	0.96
64 × 64	**0.971**	0.988	0.965
72 × 72	0.964	0.988	0.969
128 × 128	0.963	0.987	**0.974**
Average value	0.82	**0.99**	0.971

**Table 6:** The results under optimal threshold.

Category	Quantity Statistics				Performance Indicators			FPS
	TN	FN	FP	TP	Recall	Precision	F1 Score	
Positioning clamp nuts	297	28	3	97	77.6	97.0	86.2	25.9
U-shaped hoop nuts	294	6	6	140	95.9	95.9	95.9	26.7
Cotter pins	261	5	39	168	97.1	81.2	88.5	26.9

**Table 7:** Results of the ablation experiments.

Methods	Category	Recall	Precision	F1-Score	AUROC	FPS
(-) VSS block	Positioning clamp nuts	0.765	0.905	0.829	0.954	34.43
	U-shaped hoop nuts	0.909	0.911	0.91	0.965	35.65
	Cotter pins	0.928	0.859	0.892	0.958	35.87
(-) CBAM	Positioning clamp nuts	0.782	0.917	0.844	0.961	29.36
	U-shaped hoop nuts	0.929	0.927	0.928	0.977	30.33
	Cotter pins	0.947	0.879	0.912	0.964	30.89
(-) None	Positioning clamp nuts	0.803	0.937	0.865	0.972	25.96
	U-shaped hoop nuts	0.94	0.94	0.94	0.991	26.76
	Cotter pins	0.962	0.901	0.931	0.975	26.95

## Data Availability

The raw data supporting the conclusions of this article will be made available by the authors on request.

## Abbreviations

CSCs	Catenary Support Components
SSM	State Space Model
VSM-UNet	Visual State Space Reconstruction Network
VSS block	Visual State Space Block
POFT	Phase-only Fourier Transform
OC-SVM	One-Class Support Vector Machines
SVDD	Support Vector Data Description
Deep-SVDD	Deep Support Vector Data Description
OOD	Out-of-Distribution Detection
AE	Autoencoder
GAN	Generative Adversarial Network
CNNs	Convolutional Neural Networks
CBAM	Convolutional Block Attention Module
ODE	Ordinary Differential Equation
CSM	Cross-Scan Module
MLP	Multi-Layer Perceptron
LN	Layer Normalization
MSE	Mean Square Error
AUROC	Area Under the Receiver Operation Feature Curve
ROC	Receiver Operation Feature Curve
FP	False Positive
TP	True Positive
SE	SE block
NL	Non-Local
DA	Dual-Attention
